# Simple functional assessment at hospital discharge can predict long-term outcomes of ICU survivors

**DOI:** 10.1371/journal.pone.0214602

**Published:** 2019-04-04

**Authors:** Ryoung-Eun Ko, Hyun Lee, Jin Hee Jung, Hee Og Lee, Insuk Sohn, Heejin Yoo, Jin Yeong Ko, Gee Young Suh, Chi Ryang Chung

**Affiliations:** 1 Department of Critical Care Medicine, Samsung Medical Center, Sungkyunkwan University School of Medicine, Seoul, South Korea; 2 Division of Pulmonary Medicine and Allergy, Department of Internal Medicine, Hanyang University College of Medicine, Seoul, South Korea; 3 Advanced Practice Nurse, Department of Nursing, Samsung Medical Center, Seoul, South Korea; 4 Statistics and Data Center, Samsung Medical Center, Seoul, South Korea; 5 Department of pharmaceutical services, Samsung Medical Center, Seoul, South Korea; 6 Division of Pulmonology and Critical Care Medicine, Department of Medicine, Samsung Medical Center, Sungkyunkwan University School of Medicine, Seoul, Korea; 7 Department of Medicine, Samsung Medical Center, Sungkyunkwan University School of Medicine, Seoul, Korea; Azienda Ospedaliero Universitaria Careggi, ITALY

## Abstract

Recent studies showed that physical and/or neuropsychiatric impairments significantly affect long-term mortality of ICU survivors. We conducted this study to investigate that simplified measurement of physical function and level of consciousness at hospital discharge by attending nurses could predict long-term outcomes after hospital discharge. A retrospective analysis of prospectively and retrospectively collected data of 246 patients who received medical ICU treatment was conducted. We grouped patients according to physical function and level of consciousness measured by the simplified method at hospital discharge as follow; group A included patients with alert mental and capable of walking or moving by wheel chairs; group B included those with alert mental and bed-ridden status; and Group C included those with confused mental and bed-ridden status. The two-year survival rate after hospital discharge was compared. Of 246 patients, 157 patients were included in the analysis and there were 103 survivors after two-year follow up. Compared to non-survivors, survivors were more likely to be younger (*P* = 0.026) and have higher body mass index (*P* = 0.019) and no malignant disease (*P* = 0.001). There were no statistically significant differences in treatment modalities including medication, use of medical devices, and physical therapy between the survivors and non-survivors. The analysis showed significant differences in survival between the groups classified by physical function (*P* < 0.001) and level of consciousness (*P* < 0.01). Multivariate analysis showed that survival rate was significantly lower among the patients in group C than in those in group B or group A (*P* < 0.001). Simplified method to assess physical function and level of consciousness at hospital discharge can predict long-term outcomes of medical ICU survivors.

## Introduction

Recent advances in intensive care unit (ICU) management have reduced the mortality of patients during ICU and hospital treatment [1,2]. Although in-hospital mortality has decreased, the long-term treatment outcomes of ICU survivors are still poor [3,4]. Thus, increasing interest has been shown in finding clinical factors that can predict the long-term mortality of ICU survivors [5,6].

ICU survivors often experience persistent physical and/or neuropsychiatric impairments, known together as post-intensive care syndrome [7–9]. Numerous studies have shown that these impairments are not only long-lasting but are also significantly associated with long-term prognosis [10–14]. Reflecting the importance of these factors on the long-term outcomes of ICU survivors, the National Institutes of Health NHLBI ARDS Network proposed the endotyping method to advance understanding of post ICU care patients according to physical, cognitive, and mental health status by showing that decreased physical function with decreased mental health was related to poor long-term outcomes of ICU survivors [15]. However, despite the clinical relevance of this study, very complicated scales were applied that are time-consuming and need specialized teams of physical therapists or specialists, precluding the potential use of the endotyping method in screening programs on patients in real-clinics.

Therefore, it is reasonable to suggest that a more convenient and simplified method could be performed during daily practice to predict the long-term mortality of ICU-survivors. In our institution, the physical function and level of consciousness of the patients are mandatorily evaluated by the attending nurses at hospital discharge. However, no previous study has attempted to evaluate whether this simplified functional assessment at hospital discharge can predict long-term morality of ICU-survivors. Using a prospective observational medical ICU patients data for quality improvement (QI) in nursing and retrospectively supplemented for analysis, we investigated whether simplified measurement of physical function and level of consciousness at hospital discharge by attending nurses could predict long-term outcomes after hospital discharge in medical ICU patients.

## Materials and methods

### Study patients

**Fig 1:**
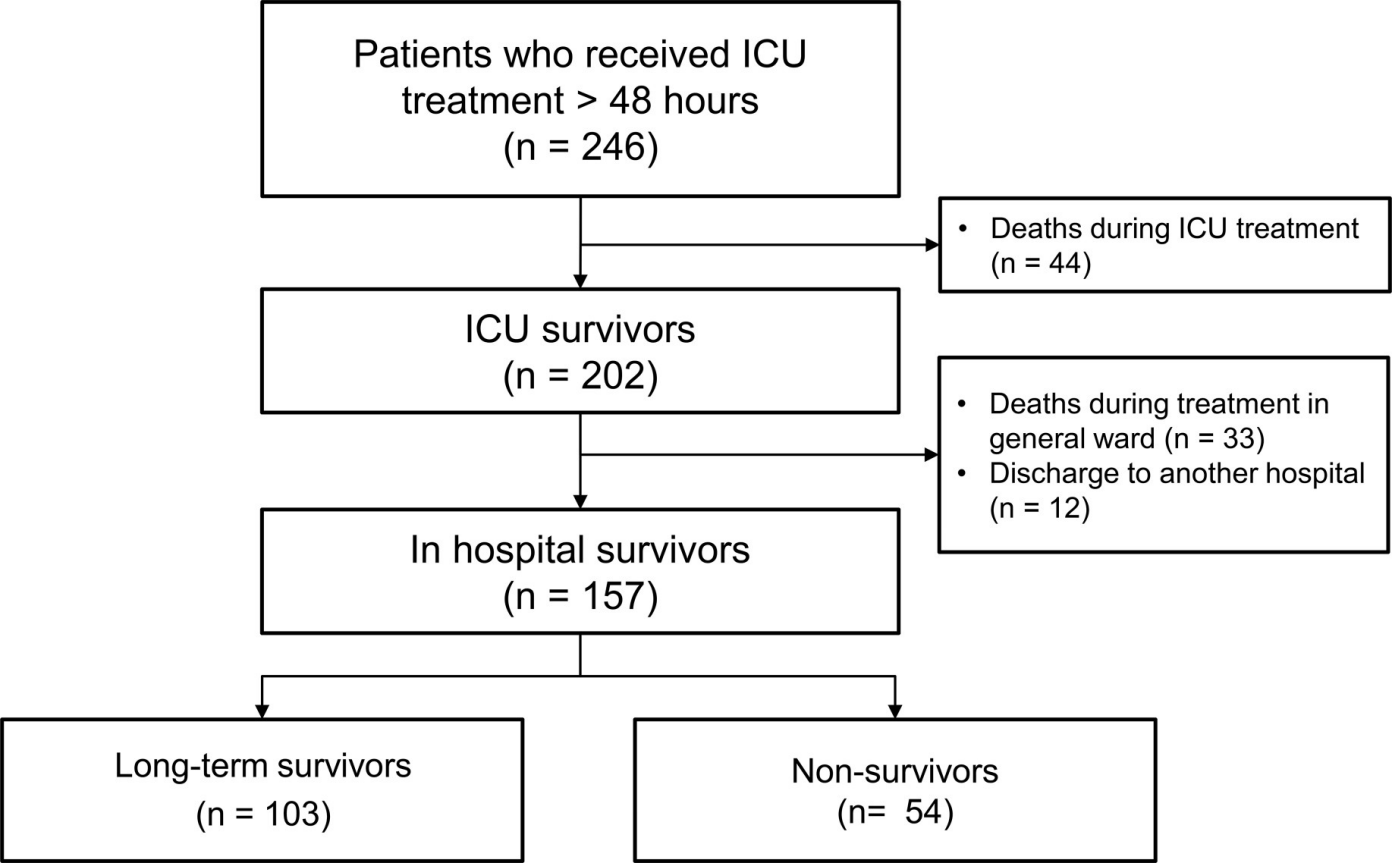
Flow chart.

Data of 246 patients who were admitted to the medical ICU at the Samsung Medical Center (a 1961-bed, university-affiliated, tertiary referral hospital in Seoul, Korea) for at least 48 hours and recorded for QI in nursing between December 2013 and August 2014 was conducted. Of the 246 patients, 77 died before hospital discharge and 12 were excluded since they were admitted to another hospital at hospital discharge. Finally, 157 patients were included in this study (Fig 1).

### Data collection

Data were prospectively collected for the quality improvement (QI) project of the ICU to improve the quality of the intensive care unit, and additional variables from the relevant data retrospectively collected through medical records.

Medical records were collected from each patient at three different stages: at the time of ICU admission, during ICU care, and at hospital discharge. At the time of ICU admission, data including age, gender, body mass index (BMI), smoking history, previous medical history, reason for admission to ICU, activity before admission, admission source (ward, emergency room, other ICU within hospital or outside hospital), and Simplified Acute Physiology Score (SAPS) 3 were evaluated. During ICU care, the medical data included the length of ICU stay, mobility, sedation scores, use of sedatives or analgesics, and use of artificial devices were evaluated. We further collected medical records including physical function and level of consciousness at hospital discharge.

### Definitions

Long-term survival was analyzed based on mortality at two-years after hospital discharge. Dosages of opioids including fentanyl, morphine, hydromorphone, and remifentanil were converted to their fentanyl equivalent [16]. Dosages of benzodiazepines were described as midazolam dosage because we only used midazolam. To evaluate the physical function and level of consciousness at the time of hospital discharge, attending nurses prospectively assessed these factors at the time of hospital discharge, classified the patients into alert and non-alert (drowsy, stupor, and semi-coma) groups according to level of consciousness and groups who can walk, those who can move using a wheelchair, and bed-ridden groups according to locomotion function. For analyses, patients were further classified into three groups according to the composite of physical function and level of consciousness as follows: Group A included patients who were alert and capable of walking or moving using a wheelchair; Group B included patients who were alert, but in bed-ridden status; and Group C included patients who were not alert and in bed-ridden status. Delirium was assessed three times a day using the Confusion Assessment Method-ICU (CAM-ICU) [17]. For analyses, we calculated the area under the curve (AUC) of CAM, which was defined as the area of the CAM value over time. Richmond Agitation-Sedation Scale (RASS) was used to estimate levels of sedation and agitation and were checked once daily [18]. Each RASS measurement was classified into favorable (-1, 0, 1) and unfavorable (-5, -4, -3, -2, 2, 3, 4) measurements. For analysis, the proportion of favorable RASS measurement over a total number of RASS measurements was calculated for each patient.

### Statistical analysis

Categorical variables were analyzed using the Pearson χ2 test or Fisher exact test. Continuous variables were analyzed using the Mann-Whitney-Wilcoxon U test. Multivariate analysis using the Cox-regression model was used to evaluate whether the composite mental and performance status are significantly associated with long-term survival after hospital discharge. Initial candidate variables were those at the time of ICU admissions (including age, sex, BMI, SAPS3, the presence of malignancy, use of mechanical ventilation, RASS, and CAM) and those at the time of hospital discharge (including the level of consciousness and functional status). Covariates with P < 0.2 in univariate analysis, a variance inflation factor of less than 4, and those clinically relevant were entered into the multivariate model. All tests were two-sided, and a P value of less than 0.05 was considered statistically significant. All analyses were performed using SPSS for Windows (ver. 24.0; IBM Corp., Armonk, NY, USA) and R version 3.2.3 (R Foundation for Statistical Computing, Vienna, Austria)

### Ethics approval and consent to participate

This study was a retrospective observational study and the study protocol was approved by the institutional review board of the Samsung Medical Center to review and publish information obtained from patient records (IRB no. 2018-01-021-002). Informed consent was waived by the IRB due to the retrospective study design. All methods employed in this study were performed in accordance with the relevant guidelines and regulations.

## Results

### Baseline characteristics

The baseline characteristics of 157 patients are described in Table 1. Of the 157 patients, the median age was 66.0 years (interquartile range [IQR], 54.0–75.0 years) and 99 (63.1%) were men. The median BMI was 22.5 kg/m^2^ (IQR, 20.4–25.1 kg/m^2^). Common previous medical histories were hypertension (n = 85, 54.1%), diabetes mellitus (n = 51, 32.5%), and malignancy (n = 34, 21.7%). The common causes of ICU admission were respiratory failure (n = 81, 51.6%) and sepsis (n = 35, 22.3%). Regarding physical activity before ICU admission, 103 patients (65.6%) were independent. Seventy-seven patients (49.0%) were admitted through the emergency room and 53 patients (33.8%) were referred from the general ward within the hospital. The median SAPS 3 score and the Acute Physiology and Chronic Health Evaluation II score (APACHE II) were 51.0 (IQR, 40.0–62.0) and 23.0 (IQR, 17.5–30.0), respectively.

**Table 1:** Baseline characteristics of study population.

	Total (*N* = 157)	Survivors (*n* = 103)	Non-survivors (*n* = 54)	*p* value
Age, years	66.0 (54.0–75.0)	64.0 (51.5–72.5)	69.5 (58.0–76.0)	0.026
Gender, Male	99 (63.1)	62 (60.2)	37 (68.5)	0.394
BMI,kg/m^2^	22.5 (20.4–25.1)	22.9 (20.9–25.5)	21.4 (19.6–24.2)	0.019
Ever smoker	15 (9.6)	10 (9.7)	5 (9.3)	>0.999
Previous medical history				
Hypertension	85 (54.1)	54 (52.4)	31 (57.4)	0.670
Diabetes mellitus	51 (32.5)	37 (35.9)	14 (25.9)	0.275
Cerebrovascular disease	15 (9.6)	8 (7.8)	7 (13.0)	0.444
Malignancy	34 (21.7)	14 (13.6)	20 (37.0)	0.001
Chronic kidney disease	24 (15.3)	18 (17.5)	6 (11.1)	0.413
ICU admission diagnosis				0.468
Respiratory	81 (51.6)	51 (49.5)	30 (55.6)	
Cardiovascular	11 (7.0)	5 (4.9)	6 (11.1)	
Sepsis	35 (22.3)	25 (24.3)	10 (18.5)	
Bleeding	7 (4.5)	5 (4.9)	2 (3.7)	
Others	23 (14.6)	17 (16.5)	6 (11.1)	
Activity status before admission				0.724
Independent	103 (65.6)	68 (66.0)	35 (64.8)	
Stand by assist/supervision	15 (9.6)	10 (9.7)	5 (9.3)	
Minimal assist	9 (5.7)	4 (3.9)	5 (9.3)	
Moderate assist	17 (10.8)	12 (11.7)	5 (9.3)	
Total assist	13 (8.3)	9 (8.7)	4 (7.4)	
ICU admission source				0.557
General ward	53 (33.8)	36 (35.0)	17 (31.5)	
Emergency room	77 (49.0)	48 (46.6)	29 (53.7)	
Other hospital	14 (8.9)	11 (10.7)	3 (5.6)	
Other ICU in the hospital	10 (6.4)	7 (6.8)	3 (5.6)	
Operation room	3 (1.9)	1 (1.0)	2 (3.7)	
GCS score	14.0 (10.0–15.0)	15.0 (10.0–15.0)	14.0 (10.0–15.0)	0.457
SAPS 3 score	51.0 (40.0–62.0)	50.0 (40.0–59.0)	52.5 (43.0–66.0)	0.065
APACHE II score	23.0 (17.5–30.0)	22.5 (18.0–29.0)	25.5 (17.0–31.0)	0.341

Data are presented as median (IQR) or number (%).

BMI, body mass index; GCS, Glasgow Coma Scale; SAPS 3, Simplified Acute Physiology Score 3; APACHE II score, Acute Physiology and Chronic Health Evaluation II score.

Compared to survivors, non-survivors were more likely to be older (median 64.0 years [IQR, 51.5–72.5 years] vs. 69.5 years [IQR, 58.0–76.0 years]; *p* = 0.026), have lower BMI (median 22.9 kg/m^2^ (IQR, 20.9–25.5 kg/m^2^) vs. 21.4 kg/m^2^ (IQR, 19.6–24.2 kg/m^2^); *p* = 0.019), and have malignancy as a comorbidity (37.0% [20/54] vs. 13.6% [14/103]; *p* = 0.001). No significant differences were observed in gender, smoking history, ICU admission diagnosis, activity status before admission, ICU admission source, SAPS 3, and APACHE II between the patients who survived and non-survived after 2-year of follow up.

### Comparison of treatment modalities and sedation and delirium between survivors and non-survivors

As shown in Table 2, no statistically significant differences were found in the treatment modalities including medication (opioids, benzodiazepine, propofol, dexmedetomidine, and neuromuscular blocking agent), use of medical devices (artificial airway, home ventilator, extra-corporeal membrane oxygenation, and hemodialysis), and physical therapy between the survivors and non-survivors.

**Table 2:** Comparison of treatment modalities, and of sedation and delirium between the survivors and non-survivors.

	Total (*N* = 157)	Survivors (*n* = 103)	Non-survivors (*n* = 54)	*p* value
**Medication**				
** Opioids**	63 (40.1)	41 (40.2)	22 (40.0)	>.999
** Total dose/patient/day, mcg**	3667.1 ± 24665.2	2226.2 ± 7189.5	6415.7 ± 40981.3	. 459
** Benzodiazepine infusions**	5 (3.2)	1 (1.0)	4 (7.3)	.096
** Total dose/patient/day, mcg**	0.9 ± 5.9	0.1 ± 1.2	2.3 ± 9.8	.105
** Propofol infusions**	17 (10.8)	9 (8.8)	8 (14.5)	.406
** Total dose/patient/day, mcg**	30.4 ± 173.6	14.1 ± 81.8	61.4 ± 272.7	.218
** Dexmedetomidine infusions**	19 (12.1)	13 (12.7)	6 (10.9)	.936
** Total dose/patient/day, mcg**	9.9 ± 36.5	11.4 ± 40.7	7.0 ± 26.7	. 415
** NMBA**	11 (7.0)	7 (6.8)	4 (7.4)	>.999
**Medical devices**				
** Artificial airway**	90 (57.3)	58 (56.3)	32 (59.3)	.853
** Mechanical ventilator**	89 (56.7)	59 (57.3)	30 (55.6)	.970
** ECMO**	7 (4.5)	4 (3.9)	3 (5.6)	.940
** Hemodialysis**	33 (21.0)	24 (23.3)	9 (16.7)	.445
**Physical therapy**	100 (63.7)	70 (68.0)	30 (55.6)	.174
**Sedation and Delirium**				
**Favorable RASS measurement**	94.9 (71.4–100.0)	95.5 (78.2–100.0)	94.4 (66.7–100.0)	.299
**Area of CAM**	1.5 (0.0–4.5)	1.0 (0.0–4.8)	1.5 (0.0–4.0)	.546
**Length of ICU stay**	4.1 (2.1–8.9)	4.3 (2.7–8.4)	3.7 (2.0–8.9)	.574

Data are presented as median and IQR or number (%).

PT, physical therapy; RASS, Richmond Agitation-Sedation Scale; CAM-ICU, Confusion Assessment Method-ICU; NMBA, neuromuscular blocker agent; ECMO, extra-corporeal membrane oxygenation.

### Comparison of mental and locomotion function at hospital discharge between survivors and non-survivors

Regarding the level of consciousness at hospital discharge, survivors were more likely to be alert than non-survivors (93.2% [96/103] vs. 81.5% [44/54]). Regarding locomotion function at hospital discharge, non-survivors were more likely to be bed-ridden than survivors (48.1% [26/54] vs. 22.3% [23/103]; *p* < 0.001).

### Kaplan-Meier analysis for 2-year survival according to mental and locomotion function

**Fig 2:**
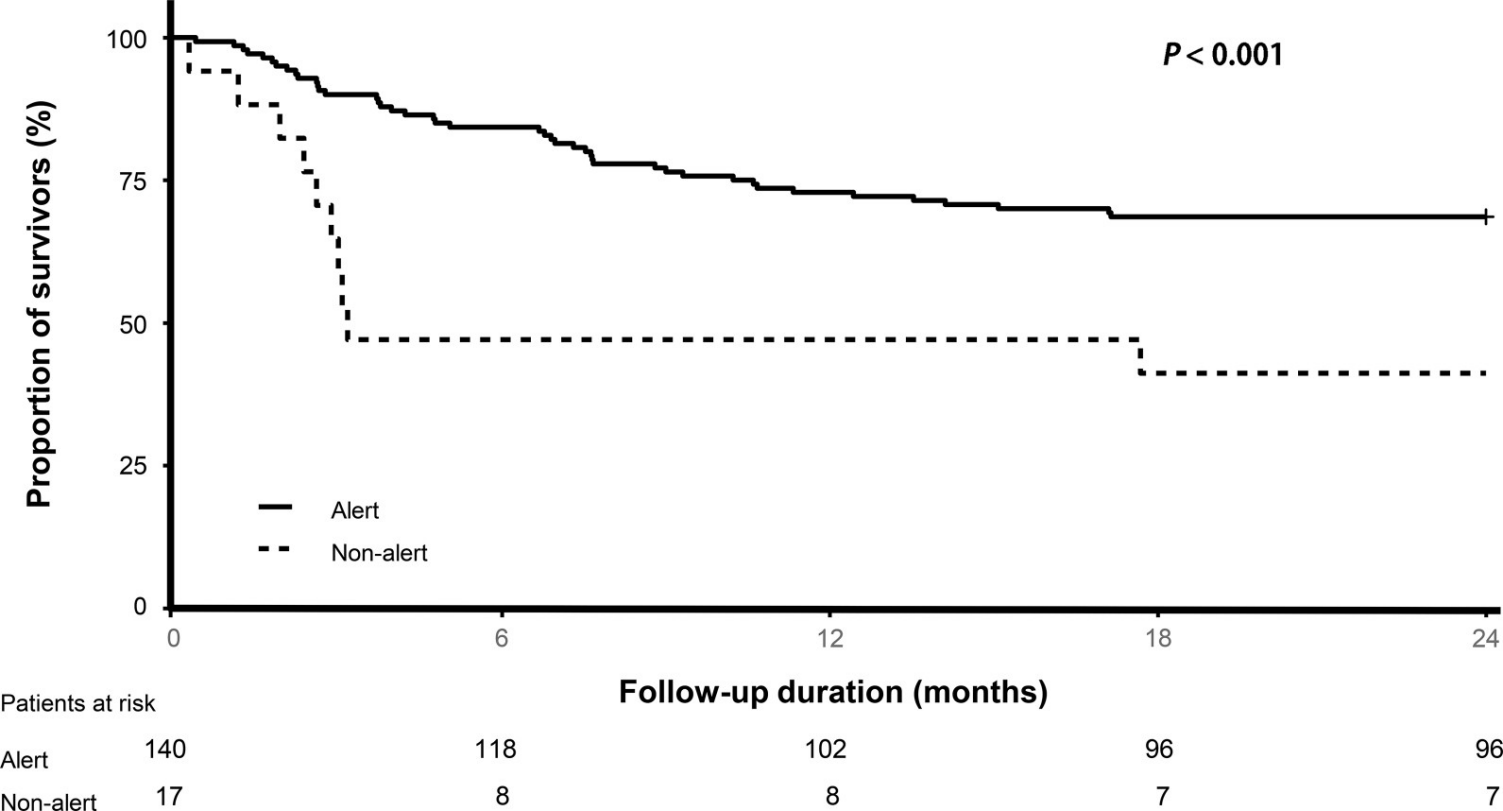
Long-term survival outcomes. Kaplan survival curve according to the level of consciousness.

**Fig 3:**
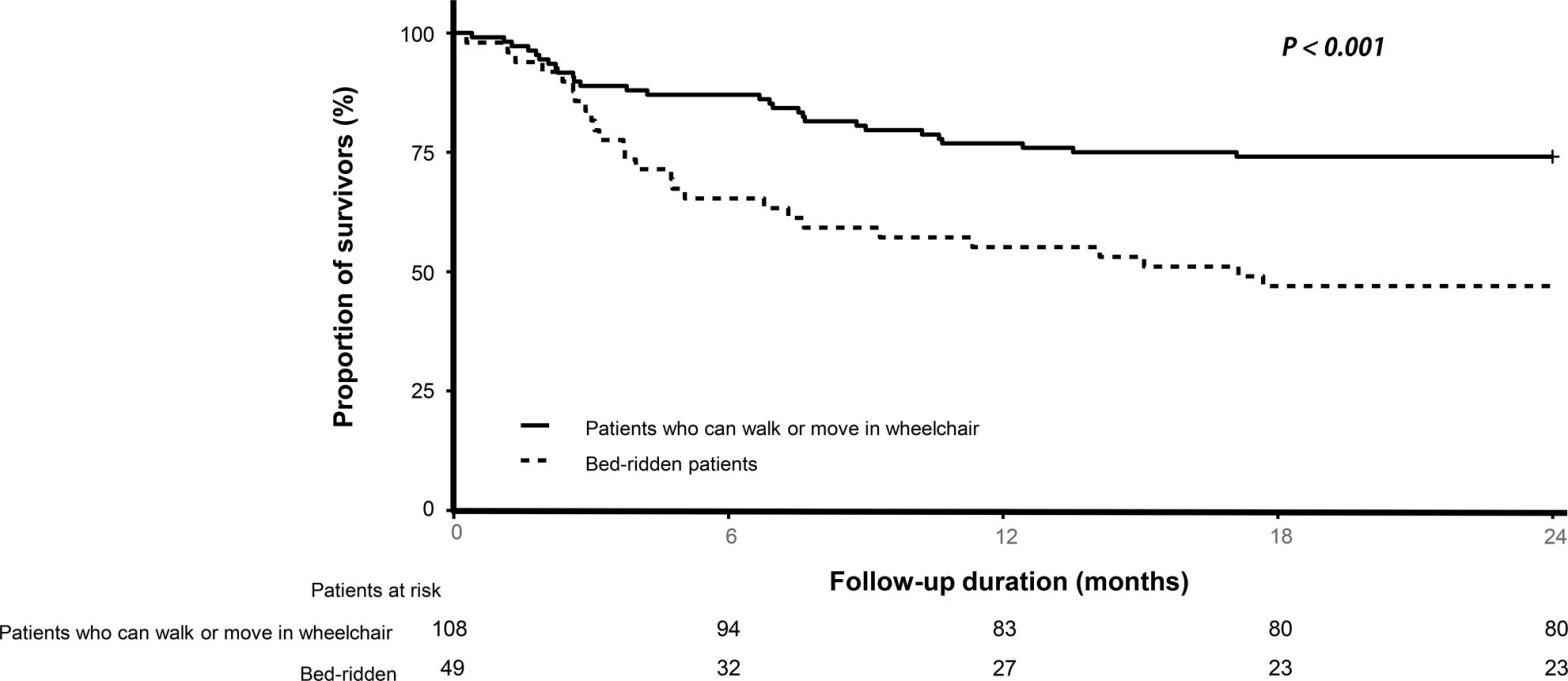
Long-term survival outcomes. Kaplan survival curve according to physical function.

The Kaplan-Meier plot showed significant differences in survival between the groups classified by level of consciousness (*p* < 0.01 for alert and non-alert groups, Fig 2) and locomotion function (*p* < 0.001 for groups who can walk, those who can move using a wheelchair, and the bed-ridden group, Fig 3).

### Clinical factors associated with survivors after 2-year follow up

**Fig 4:**
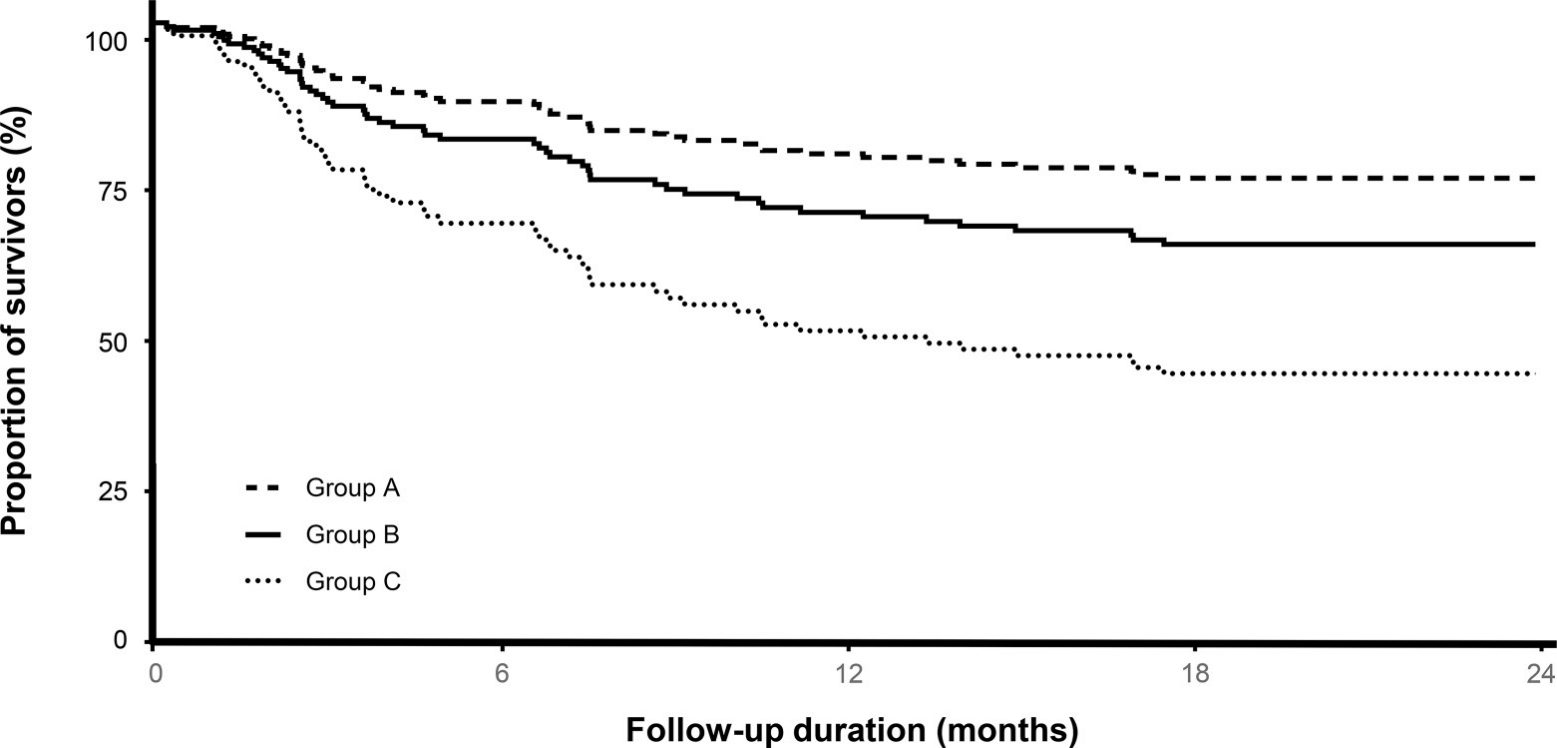
Adjusted survival curve for the Cox proportional hazard model.

In the univariate Cox proportional hazard regression analysis, age (hazard ratio [HR] = 1.02, *p* = 0.025), BMI (HR = 0.92, *p* = 0.022), SAPS 3 (HR = 1.02, HR = 0.022), the presence of malignancy (HR = 2.83, *p* < 0.001), groups according to the level of consciousness and locomotion function (group B [HR = 2.00, *p* = 0.027] and group C [HR = 3.28, *p* = 0.001] were factors associated with long-term mortality. In multivariate analyses, the presence of malignancy (adjusted HR = 2.48, *p* = 0.003), the proportion of unfavorable RASS measurement (adjusted HR = 1.09, *p* = 0.011), and group C (HR = 2.62, *p* = 0.013) were significantly associated with long-term mortality. As shown in Fig 4, significant differences were shown in the adjusted survival curves for the Cox proportional hazard model between the groups (Table 3).

**Table 3:** Clinical factors affecting long-term mortality in patients receiving ICU treatment.

	Univariate		Multivariate	
	Crude OR (95% CI)	*p*-value	Adjusted HR (95%CI)^a^	*p*-value
**Age**	1.02 (1.00–1.04)	0.025		
**Gender, Male**	0.72 (0.41–1.28)	0.264		
**BMI**	0.92 (0.86–1.00)	0.022	0.94 (0.87–1.01)	0.085
**SAPS 3**	1.02 (1.00–1.04)	0.035		
**The presence malignancy**	2.83 (1.63–4.93)	< 0.001	2.48 (1.36–4.52)	0.003
**Mechanical ventilation**	0.94 (0.55–1.61)	0.817		
**RASS**				
** Favorable RASS measurement**	Ref		Ref	
** Unfavorable RASS measurement**	1.01 (1.00–1.02)	0.110	1.09 (1.02–1.16)	0.011
**Area or curve of CAM-ICU**	1.02 (0.98–1.06)	0.315	0.92 (0.83–1.01)	0.087
**Groups by locomotion and mental function**				
**Group A**^**b**^	Ref		Ref	
**Group B**^**c**^	2.00 (1.08–3.70)	0.027	1.56 (0.79–3.07)	0.196
**Group C**^**d**^	3.28 (1.59–6.76)	0.001	2.62 (1.22–5.63)	0.013

BMI, body mass index; SAPS 3, Simplified Acute Physiology Score 3; CAM-ICU, Confusion Assessment Method-ICU.

^a^Adjusted for gender, age, factors with a *p* < 0.2 (BMI, SAPS3, previous malignancy, group), and mechanical ventilator, RASS, area of CAM-ICU as clinically relevant.

^**b**^Group A included patients with alert mental status who were able to walk or move using a wheelchair.

^**c**^Group B included patients with alert mental status but were bedridden.

^**d**^Group C included patients with non-alert mental status and bedridden.

## Discussion

In this study, the relationship between physical function and level of consciousness was evaluated at hospital discharge and subsequent long-term mortality. Our study showed that a simple assessment of physical function or level of consciousness by attending nurses at hospital discharge can predict the long-term mortality of medical ICU survivors. Compared to the patients without physical or cognitive abnormality, those who had both decreased physical function and impaired consciousness had a greater incidence of long-term mortality. To the best of our knowledge, this is the first study showing the effectiveness of simple functional assessment on predicting long-term outcomes of medical ICU survivors.

In this study, during the two-year follow-up duration, about a third of the patients who had received critical care died after hospital discharge; this is consistent with the previous studies which revealed that the long-term survival rate of ICU survivors is still suboptimal [19–21]. Surprisingly, more than half of the ICU survivors had functional abnormalities associated with being incapable of unassisted walking, and more than 10% of ICU survivors had both impaired functional abnormalities and level of consciousness [4]. These results suggest that an augmented strategy to improve physical and mental health is urgently required to improve the long-term outcomes of ICU survivors.

Our study revealed that functional impairment at hospital discharge is an independent factor for predicting long-term outcomes of ICU survivors. This finding is consistent with findings shown in previous studies that explored the association between physical or neuropsychiatric functions and long-term prognosis in ICU survivors.

Extending the findings of previous studies, our study showed a combined effect of physical function and level of consciousness on long-term treatment outcomes of ICU survivors. Interestingly, after adjusting for confounding factors, the sole presence of physical impairment was not a solid criterion for discrimination between survivors and non-survivors. However, the additional presence of an impaired level of consciousness independently predicted the poor outcomes in ICU survivors. These results might suggest that clinicians need to pay more attention to those subjects with physical impairments, but with intact consciousness since these factors might be adjusted by active treatment such as rehabilitation. However, since we have no data regarding this issue, further studies are needed to confirm our suggestions.

Another strength of our study is the simple functional assessment performed by attending nurses. While various methods have been proposed of measuring physical and neuropsychiatric function that can accurately predict long-term treatment outcomes of ICU survivors [7,22], most of these have been calculated through complex formulas, which might not be applicable to most hospitals in real-world clinic since most ICUs suffer from insufficient medical resources, including medical staff [23,24]. Therefore, it is very difficult to apply these complex formulas in real clinical ICU. In this view, our results provide informative data whereby long-term mortality can be predicted by a simple assessment, measuring physical function according to locomotion function (such as the ability to walk or move using a wheelchair, and bed-ridden status) and level of consciousness (arousal or non-arousal). This approach might be more suitable to real-world ICU settings. And, the simple functional assessment is applicable to patient by not only nurses but also all medical staff. Therefore, it might be useful to set an easy and clear goal of discharge patients by every medical staff. Caregivers also can easily understand the long-term prognosis of the patient through easily understandable assessment method, so the physician can discuss the plan according to the patient’s progress and reduce unnecessary ICU re-admission.

This study has several limitations. First, this study was conducted at a single center medical ICU in South Korea, and the simple assessment performed might therefore not be applicable to patients in other ICUs in other countries. Thus, further studies are needed to confirm our methods. Second, due to the observational design, our study might have inherent biases related to confounding, potential reverse causation, and the lack of a randomly distributed exposure. Third, we were unable to adjust for psychiatric variables, mental status and level of activity before admission that might have affected physical function and level of consciousness. Forth, only the history of cerebral vascular disease and GCS score at admission were included in the patient’s neurological baseline characteristics.

## Conclusion

Our study showed that a simple assessment of physical function and level of consciousness at hospital discharge can predict long-term mortality of medical ICU survivors, which suggests the need for augmented treatment strategies to improve physical function and level of consciousness in ICU care. However, since this method has not been validated in other cohorts, a validation study is needed.

## Supporting information

S1 Table Comparison of mental and locomotion functions between survivors and non-survivors. (DOCX)

S1 Dataset PONE-D-18-33786R1_FTC_rawdata.csv. (CSV)

## References

[1] Zambon M, Vincent JL. Mortality rates for patients with acute lung injury/ARDS have decreased over time. Chest. 2008; 133:1120–7. 10.1378/chest.07-2134 18263687

[2] Erickson SE, Martin GS, Davis JL, Matthay MA, Eisner MD, Network NNA. Recent trends in acute lung injury mortality: 1996–2005. Crit Care Med. 2009; 37:1574–9. 10.1097/CCM.0b013e31819fefdf 19325464 PMC2696257

[3] Stevens RD, Hart N, Herridge MS. Textbook of Post-ICU Medicine, The Legacy of Critical Care Oxford: Oxford University Press; 2014.

[4] Hashem MD, Nallagangula A, Nalamalapu S, Nunna K, Nausran U, Robinson KA, et al Patient outcomes after critical illness: a systematic review of qualitative studies following hospital discharge. Crit Care. 2016; 20:345 10.1186/s13054-016-1516-x 27782830 PMC5080744

[5] Tipping CJ, Harrold M, Holland A, Romero L, Nisbet T, Hodgson CL. The effects of active mobilisation and rehabilitation in ICU on mortality and function: a systematic review. Intensive Care Med. 2017; 43:171–83. 10.1007/s00134-016-4612-0 27864615

[6] Parry SM, Knight LD, Connolly B, Baldwin C, Puthucheary Z, Morris P, et al Factors influencing physical activity and rehabilitation in survivors of critical illness: a systematic review of quantitative and qualitative studies. Intensive Care Med. 2017; 43:531–42. 10.1007/s00134-017-4685-4 28210771

[7] Herridge MS, Tansey CM, Matte A, Tomlinson G, Diaz-Granados N, Cooper A, et al Functional disability 5 years after acute respiratory distress syndrome. N Engl J Med. 2011; 364:1293–304. 10.1056/NEJMoa1011802 21470008

[8] Dowdy DW, Eid MP, Sedrakyan A, Mendez-Tellez PA, Pronovost PJ, Herridge MS, et al Quality of life in adult survivors of critical illness: a systematic review of the literature. Intensive Care Med. 2005; 31:611–20. 10.1007/s00134-005-2592-6 15803303

[9] Desai SV, Law TJ, Needham DM. Long-term complications of critical care. Crit Care Med. 2011; 39:371–9. 10.1097/CCM.0b013e3181fd66e5 20959786

[10] Rydingsward JE, Horkan CM, Mogensen KM, Quraishi SA, Amrein K, Christopher KB. Functional Status in ICU Survivors and Out of Hospital Outcomes: A Cohort Study. Crit Care Med. 2016; 44:869–79. 10.1097/CCM.0000000000001627 26929191 PMC4833588

[11] Neumeier A, Nordon-Craft A, Malone D, Schenkman M, Clark B, Moss M. Prolonged acute care and post-acute care admission and recovery of physical function in survivors of acute respiratory failure: a secondary analysis of a randomized controlled trial. Crit Care. 2017; 21:190 10.1186/s13054-017-1791-1 28732512 PMC5521116

[12] Honselmann KC, Buthut F, Heuwer B, Karadag S, Sayk F, Kurowski V, et al Long-term mortality and quality of life in intensive care patients treated for pneumonia and/or sepsis: Predictors of mortality and quality of life in patients with sepsis/pneumonia. J Crit Care. 2015; 30:721–6. 10.1016/j.jcrc.2015.03.009 25818842

[13] Mitchell ML, Shum DHK, Mihala G, Murfield JE, Aitken LM. Long-term cognitive impairment and delirium in intensive care: A prospective cohort study. Aust Crit Care. 2018; 31:204–11. 10.1016/j.aucc.2017.07.002 28736089

[14] Baldwin MR, Reid MC, Westlake AA, Rowe JW, Granieri EC, Wunsch H, et al The feasibility of measuring frailty to predict disability and mortality in older medical intensive care unit survivors. J Crit Care. 2014; 29:401–8. 10.1016/j.jcrc.2013.12.019 24559575 PMC4012557

[15] Brown SM, Wilson EL, Presson AP, Dinglas VD, Greene T, Hopkins RO, et al Understanding patient outcomes after acute respiratory distress syndrome: identifying subtypes of physical, cognitive and mental health outcomes. Thorax. 2017; 72:1094–103. 10.1136/thoraxjnl-2017-210337 28778920 PMC5690818

[16] Principles of analgesic use in the treatment of acute pain and chronic cancer pain, 2nd edition. American Pain Society. Clin Pharm. 1990; 9:601–12. 2201478

[17] Ely EW, Margolin R, Francis J, May L, Truman B, Dittus R, et al Evaluation of delirium in critically ill patients: validation of the Confusion Assessment Method for the Intensive Care Unit (CAM-ICU). Crit Care Med. 2001; 29:1370–9. 11445689 10.1097/00003246-200107000-00012

[18] Ely EW, Truman B, Shintani A, Thomason JW, Wheeler AP, Gordon S, et al Monitoring sedation status over time in ICU patients: reliability and validity of the Richmond Agitation-Sedation Scale (RASS). JAMA. 2003; 289:2983–91. 10.1001/jama.289.22.2983 12799407

[19] Brinkman S, de Jonge E, Abu-Hanna A, Arbous MS, de Lange DW, de Keizer NF. Mortality after hospital discharge in ICU patients. Crit Care Med. 2013; 41:1229–36. 10.1097/CCM.0b013e31827ca4e1 23591209

[20] Wunsch H, Guerra C, Barnato AE, Angus DC, Li G, Linde-Zwirble WT. Three-year outcomes for Medicare beneficiaries who survive intensive care. JAMA. 2010; 303:849–56. 10.1001/jama.2010.216 20197531

[21] Winters BD, Eberlein M, Leung J, Needham DM, Pronovost PJ, Sevransky JE. Long-term mortality and quality of life in sepsis: a systematic review. Crit Care Med. 2010; 38:1276–83. 10.1097/CCM.0b013e3181d8cc1d 20308885

[22] Hopkins RO, Weaver LK, Collingridge D, Parkinson RB, Chan KJ, Orme JF Jr. Two-year cognitive, emotional, and quality-of-life outcomes in acute respiratory distress syndrome. Am J Respir Crit Care Med. 2005; 171:340–7. 10.1164/rccm.200406-763OC 15542793

[23] Lee H, Ko YJ, Suh GY, Yang JH, Park CM, Jeon K, et al Safety profile and feasibility of early physical therapy and mobility for critically ill patients in the medical intensive care unit: Beginning experiences in Korea. J Crit Care. 2015; 30:673–7. 10.1016/j.jcrc.2015.04.012 25957499

[24] Du B, Xi X, Chen D, Peng J, China Critical Care Clinical Trial G. Clinical review: critical care medicine in mainland China. Crit Care. 2010; 14:206 10.1186/cc8222 20236446 PMC2875498

