# Supplementary material for: Simple functional assessment at hospital discharge can predict long-term outcomes of ICU survivors
Source: PLoS One. 2019 Apr 4;14(4):e0214602. doi: 10.1371/journal.pone.0214602 (PMC6448871; doi:10.1371/journal.pone.0214602)
Supplement: S1 Table — (DOCX) [file pone.0214602.s001.docx]

**Supplementary Table 1. Comparison of Mental and Locomotion Functions Between Survivors and Non-Survivors**

| Variables | **Total (*N* = 157)** | **Survivors (*n* = 103)** | **Non-survivors (*n* = 54)** |  |
| --- | --- | --- | --- | --- |
| level of consciousness |  |  |  | 0.048 |
| Alert | 140 (89.2) | 96 (93.2) | 44 (81.5) |  |
| Not alert | 17 (10.8) | 7 (6.8) | 10 (18.5) |  |
| Locomotion function |  |  |  | 0.003 |
| Walk | 72 (45.9) | 55 (53.4) | 17 (31.5) |  |
| Move with wheel chair | 36 (22.9) | 25 (24.3) | 11 (20.4) |  |
| Bed-ridden | 49 (31.2) | 23 (22.3) | 26 (48.1) |  |
| Groups classified according to composite of level of consciousness and locomotion function |  |  |  | 0.011 |
| Group A | 72 (45.9) | 55 (53.4) | 17 (31.5) |  |
| Group B | 68 (43.3) | 41 (39.8) | 27 (50.0) |  |
| Group C | 17 (10.8) | 7 (6.8) | 10 (18.5) |  |

Data presented as n (%) unless otherwise indicated.

Group A refers to patients with alert consciousness and locomotion while walking or in a wheelchair. Group B refers to patients with alert consciousness and locomotion while in bed. Group C refers to patients with no alert consciousness and locomotion while in a bed.
